# Changes in the Small Noncoding RNAome During M1 and M2 Macrophage Polarization

**DOI:** 10.3389/fimmu.2022.799733

**Published:** 2022-05-10

**Authors:** Ding Ma, Xing Zhou, Yu Wang, Liming Dai, Jie Yuan, Jianping Peng, Xiaoling Zhang, Chuandong Wang

**Affiliations:** ^1^ Department of Orthopedic Surgery, Xinhua Hospital Affiliated to Shanghai Jiao Tong University School of Medicine (SJTUSM), Shanghai, China; ^2^ Collaborative Innovation Centre of Regenerative Medicine and Medical BioResource Development and Application Co-constructed by the Province and Ministry, Guangxi Medical University, Nanning, China; ^3^ Department of Cardiology, Shidong Hospital Affiliated to University of Shanghai for Science and Technology, Shanghai, China; ^4^ Department of Orthopaedic Surgery, The Second Hospital of Shanxi Medical University, Taiyuan, China

**Keywords:** macrophage polarization, miRNA, piRNA, snoRNA, snRNA, repeat RNA

## Abstract

Macrophages belong to a special phagocytic subgroup of human leukocytes and are one of the important cells of the human immune system. Small noncoding RNAs are a group of small RNA molecules that can be transcribed without the ability to encode proteins but could play a specific function in cells. SncRNAs mainly include microRNAs (miRNAs) and piwi-interacting RNAs (piRNAs), small nucleolar RNAs (snoRNAs), small nuclear RNAs (snRNAs) and repeat RNAs. We used high-throughput sequencing analysis and qPCR to detect the expression changes of the small noncoding RNAome during macrophage polarization. Our results showed that 84 miRNAs and 47 miRNAs with were downregulated during M1 macrophage polarization and that 11 miRNAs were upregulated and 19 miRNAs were downregulated during M2 macrophage polarization. MiR-novel-3-nature and miR-27b-5p could promote expression of TNF-α which was marker gene of M1 macrophages. The piRNA analysis results showed that 69 piRNAs were upregulated and 61 piRNAs were downregulated during M1 macrophage polarization and that 3 piRNAs were upregulated and 10 piRNAs were downregulated during M2 macrophage polarization. DQ551351 and DQ551308 could promote the mRNA expression of TNF-α and DQ551351overexpression promoted the antitumor activity of M1 macrophages. SnoRNA results showed that 62 snoRNAs were upregulated and 59 snoRNAs were downregulated during M1 macrophage polarization, whereas 6 snoRNAs were upregulated and 10 snoRNAs were downregulated during M2 macrophage polarization. Overexpression of snoRNA ENSMUST00000158683.2 could inhibit expression of TNF-α. For snRNA, we found that 12 snRNAs were upregulated and 15 snRNAs were downregulated during M1 macrophage polarization and that 2 snRNAs were upregulated during M2 macrophage polarization. ENSMUSG00000096786 could promote expression of IL-1 and iNOS and ENSMUSG00000096786 overexpression promoted the antitumor activity of M1 macrophages. Analysis of repeat RNAs showed that 7 repeat RNAs were upregulated and 9 repeat RNAs were downregulated during M1 macrophage polarization and that 2 repeat RNAs were downregulated during M2 macrophage polarization. We first reported the expression changes of piRNA, snoRNA, snRNA and repeat RNA during macrophage polarization, and preliminarily confirmed that piRNA, snoRNA and snRNA can regulate the function of macrophages.

## Introduction

Macrophages belong to a special phagocytic subgroup of human leukocytes and are one of the important cells of the human immune system (1). They participate in both specific and innate immune processes and play an important role in the host’s resistance to pathogen invasion and inflammatory response (2). Macrophage polarization is a response to different degrees of activation in a specific time and space; that is, different macrophage subtypes form in different tissues, different microenvironments and different signal stimuli, and there are mainly M1-polarized macrophages with a proinflammatory phenotype and M2-polarized macrophages with an anti-inflammatory phenotype (3). M1 polarized macrophages overexpress CD80, CD86 and CD16/32c and can produce a proinflammatory response and proinflammatory-related factors such as interleukin-6 (IL-6), interleukin-12 (IL-12) and tumor necrosis factor (TNF-α) (4). M1 macrophage polarization has been shown to be involved in the pathophysiology of many diseases, including arthritis, tumors, atherosclerosis, bacterial viral infection, diabetes, sebaceous gland disease and cryptococcal cheilitis (5). In the process of tumorigenesis and development, M1-polarized macrophages also show antitumor activity and the potential to induce tumor tissue destruction and are likely to participate in T cell-mediated tumor elimination and the balance stage. Macrophages with an M1 polarization phenotype promote the progression of RA by releasing various inflammatory cytokines (6). At the same time, M1 polarized macrophages have the potential to differentiate into mature osteoclasts, which can promote an increase in bone resorption by secreting proinflammatory cytokines and other mechanisms to mediate the occurrence of osteoporosis (7). Unlike classically activated M1 macrophages, M2 macrophages have immunomodulatory effects and highly express anti-inflammatory cytokines, including IL-10, IL-13 and transforming growth factor (TGF-β) (8). M2 macrophages could promote the regression of inflammation and immune escape of pathogens, inhibit parasites, and promote tissue repair and tumor development (9). Macrophages are a group of immune cells with high plasticity and heterogeneity that play an important role in maintaining the stability of the immune system. Under the action of different stimulating factors, macrophages can polarize into M1 and M2 types, and their polarization process is affected by a variety of signaling pathways.

Small noncoding RNAs are a group of small RNA molecules that can be transcribed without the ability to encode proteins but could play a specific function in cells (10). At present, researchers divide ncRNAs into two categories according to their length: short chain noncoding RNAs (sncRNAs) with a length of 18-200 nt and long chain noncoding RNAs (lncRNAs) with a length > 200 nt (11). Short chain noncoding RNAs are not long, but they play an important regulatory role in biological processes such as cell metabolism, proliferation, differentiation, apoptosis and immunity (12–14). The abnormal regulation of sncRNA will lead to the disorders of metabolic activities of life and the occurrence of a variety of diseases. SncRNAs mainly include microRNAs (miRNAs), piwi-interacting RNAs (piRNAs), small nucleolar RNAs (snoRNAs), small nuclear RNAs (snRNAs) and repeat RNAs (15, 16).

MiRNAs are single-stranded RNAs with a length of 20-25 nucleotides. They usually inhibit its protein synthesis and expression or leads to the degradation of mRNA by targeting the 3’-untranslated region (3’-UTR) of target mRNAs to play a posttranscriptional regulatory role (17). A miRNA can often target multiple mRNAs, and mRNAs can be regulated by a variety of miRNAs. Therefore, miRNAs can be involved in almost all aspects of biological processes, such as development, cell differentiation, intercellular communication, and immune regulation (18). miR-21 increased M2 macrophages phenotype and decreased M1 macrophages phenotype (19). miR-155 promoted M1 polarization by activating JNK pathway (20). Piwi-interacting RNA is a small RNA molecule with a length of approximately 26-31nt (21). It interacts with PIWI family proteins specifically expressed by germ cells and interacts with PIWI proteins to form the piRNA silencing complex (piRISC) (22). Compared with other known RNA family members, piRNAs showed a different set of nucleotide sequences. According to different sources, piRNAs can be divided into three categories: transposon sources, mRNA sources and lncRNA sources (23). The mature piRNA combined with PIWI can direct its complex to the transposon target of the target transcript in the nucleus and mobilize the silencing mechanism to organize the expression of transposable elements to maintain the integrity of the genome (24). To date, piRNAs have been shown to be involved in transposon silencing, epigenetic regulation, gene and protein regulation, genome rearrangement, spermatogenesis and germ cell maintenance (24, 25). Small nucleolar molecule RNA (snoRNA) is a kind of short stranded noncoding RNA related to the chemical modification of ribosomal RNA (rRNA) (26). They often serve as guides for posttranscriptional modification of rRNA. According to their sequence, structure and function, they can be divided into two categories: Box C/D snoRNA and Box H/ACA snoRNA (27). All snoRNAs are paired with specific proteins to form small nucleolar ribonucleoprotein complexes. After snoRNAs recognize and bind the complementary sequence on the target rRNA, and they send a signal to the paired protein for accurate base modification to play an important regulatory function under a variety of physiological and pathological conditions (24). Nuclear small RNA (snRNA) is a kind of small molecular RNA with a length of 100-215 nt in the eukaryotic genome that exists in the nucleus (28). SnRNA is highly conserved, the content is small in cells, and the expression is spatiotemporally specific (29). After transcription according to the gene template, snRNA is transported out of the nucleus and combined with snRNA binding protein in the cytoplasm to form small nuclear ribonucleoproteins (snRNPs), which once again pass through the nuclear pore and enter the nucleus to become mature snRNA (30). The main function of snRNA is to participate in the splicing of RNA in cells, produce mature mRNA and rRNA, and regulate protein synthesis (31). Repeat RNA is a kind of RNA containing specific repeats (32). At present, there are relatively few reports on the function of these repeat RNAs.

In this study, we used high-throughput sequencing analysis and qPCR to detect the expression changes of the small noncoding RNAome during macrophage polarization to construct the changes of the small noncoding RNAome during macrophage polarization to identify the key small RNA regulating macrophage polarization to find a new target to intervene in macrophage polarization.

## Method And Materials

### M1 and M2 Macrophage Polarization Induction

According to previous studies, we used RAW264.7 cells to induce macrophage polarization (33, 34). For M1 macrophage polarization, we added LPS (100 ng/ml)- and IFN-γ (20 ng/ml)-stimulated RAW264.7 cells for 48 hours. For macrophage M2 polarization, we stimulated RAW264.7 cells with IL-4 (20 ng/ml) and IL-10 (20 ng/ml) for 48 hours.

### Small RNA Sequencing

PCR product bands of about 140-150bp were used for small RNA sequencing. An Illumina NextSeq 550 was used for sequencing, and the sequenced original image data were converted into the original sequencing sequence. To comprehensively predict noncoding RNAs of different lengths, we first extracted the sequencing sequences of the reference genome in each sample, analyzed the sequences of all samples using cdhit, and screened the noncoding RNAs using the RFAM database. miRDeep2 were used for miRNA analysis. Blastn(2.9.0+) were used for piRNA, snoRNA and snRNA analysis. RepeatMasker were used for repeatRNA analysis. The sequencing data of each sample were compared, and the read count of small RNA of each sample was counted. The CPM of every non-coding RNAs were list in Supplementary material S1. The expression of all small RNAs in the two groups of samples was counted to judge whether there was a significant difference in the expression between the two groups of samples. For the experiments with biological repetition, we used 10 deseq for analysis. For experiments without biological duplication, we used edger for analysis. Finally, the genes with Padj less than 0.05 and a difference multiple greater than or equal to 2 or 1.5 were selected as small RNAs with significant differential expression.

### RNA Isolation and qPCR Analysis

According to the reagent instructions, macrophage RNA was extracted by TRIzol reagent, and the quality and concentration of the extracted RNA were detected by a Nanodrop2000. For the detection of miRNA, piRNA and a small part of snoRNA and snRNA, 1 μg of total RNA from all samples were extracted and reverse transcribed with miRNA first strand cDNA synthesis (tailing reaction) according to the instructions into cDNA. For the detection of most of the snoRNAs and snRNAs, 1 μg of total RNA was extracted from all samples, and PrimeScript™ RT Master Mix was used for reverse transcription into cDNA according to the instructions. QPCR detection was performed according to the instructions of the SYBR premix ex Taq Kit (RR420a, Takara, Tokyo, Japan). The relative expression value of small RNA was calculated using the 2^−ΔΔCT^ method and U6 as an internal parameter. Primers used are listed in the supplementary material S2.

### Tumor Model Establishment

The animal model was constructed according to our previous research report (35). This study was conducted in accordance with guidelines approved by the Xinhua Hospital Affiliated to Shanghai Jiao Tong University School of Medicine (approval number: XHEC-NSFC-2021-232). 8 week old male C57BL/6 mouse purchased from Shanghai Jihui experimental animal breeding Co., Ltd. RM1 cells (1×10^6^ cells) were subcutaneously injected into the right rear flanks of the mouse. There days after tumor cell inoculation, mouse treated with intratumoral injection of LPS-pretreated M1 macropahge (1×10^6^ cells) every 3 d. On 14^th^ day, all mice were euthanized, and solid tumors were surgically removed for measurements.

### Statistical Analysis

All data are shown as the mean ± S.D. of three independent tests (n≥3), and the independent sample t-tests statistical analysis was performed using Excel 2007; graphs were generated by GraphPad Prism 9. P values less than 0.05 were considered significant.

## Results

### Changes in miRNA Expression During Macrophage Polarization

**Figure 1:**
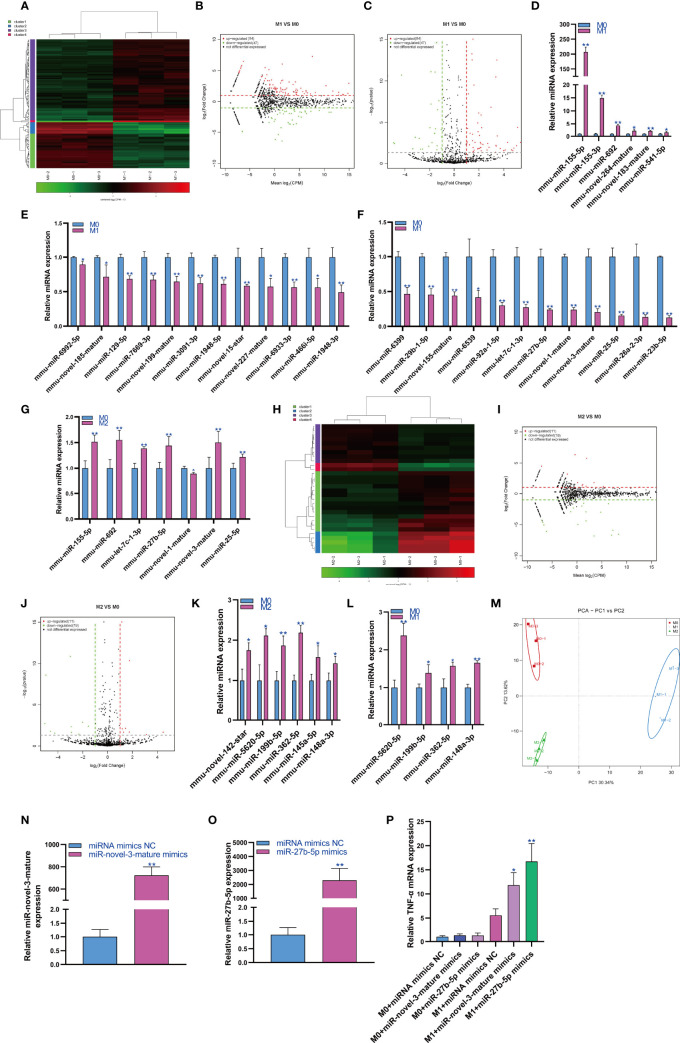
Changes in miRNA expression during macrophage polarization. Hotmap **(A)**, MA **(B)** and volcano plot **(C)** show the small RNA sequencing results of the expression of miRNA in M0 and M1 macrophages. **(D)** The expression of miRNAs upregulated in M1 macrophages detected by qPCR. **(E, F)** The expression of miRNAs downregulated in M1 macrophages detected by qPCR. **(G)** The expression of miRNA in M2 macrophages detected by qPCR. Hotmap **(H)**, MA **(I)** and volcano plot **(J)** show the small RNA sequencing results of the expression of miRNA in M0 and M2 macrophages. **(K)** The expression of miRNA in M2 macrophages detected by qPCR. **(L)** The expression of miRNA in M1 macrophages detected by qPCR. **(M)** PCA of miRNA expression in M0, M1 and M2 macrophages. * indicates p < 0.05 compared to M0, and ** indicates p < 0.01 compared to M0. **(N)** The expression of miR-novel-3-mature in RAW264.7 cells transfected with miRNA mimics NC or miR-novel-3-mature mimics were detected by qPCR. **(O)** The expression of miR-27b-5p in RAW264.7 cells transfected with miRNA mimics NC or miR-27b-5p mimics were detected by qPCR. **(P)** The expression of TNF-α in RAW264.7 cells were detected by qPCR. * indicates p < 0.05 and ** indicates p < 0.01 compared to M1+ miRNA mimics NC group.

MiRNAs play an important role in regulating cell function. Therefore, we first analyzed the expression changes of miRNAs during macrophage polarization. The results showed that 84 miRNAs were upregulated and 47 miRNAs were downregulated during M1 macrophage polarization (**Figures 1A–C** and **Figure S1**). qPCR analysis showed that 6 miRNAs, mmu-miR-155-5p, mmu-miR-155-3p, mmu-miR-692, mmu-novel-264-mature, mmu-novel-183-mature and mmu-miR-541-5p, were upregulated during M1 polarization (**Figure 1D**). On the other hand, qPCR analysis showed that 24 miRNAs, including 7 novel predicted miRNAs, were downregulated during M1 polarization (**Figures 1E, F**). Then, we detected the expression of miRNAs with significant differentiation during M1 polarization and during M2 polarization. qPCR results showed that the expressions of mmu-miR-155-5p and mmu-miR-692 were upregulated and mmu-novel-264-mature was downregulated during M2 polarization, which was consistent with M1 polarization. However, the expressions of mmu-let-7c-1-3p, mmu-miR-27b-5p, mmu-novel-3-mature and mmu-miR-25-5p were upregulated during M2 polarization, which was opposite to that during M1 polarization (**Figure 1G**). The results showed that 11 miRNAs were upregulated and 19 miRNAs were downregulated during macrophage M2 polarization (**Figures 1H–J**). qPCR analysis showed that 6 miRNAs, mmu-novel-142-star, mmu-miR-5620-5p, mmu-miR-199b-5p, mmu-miR-362-5p, mmu-miR-145a-5p and mmu-miR-148a-3p, were upregulated during M2 polarization (**Figure 1K**). However, we did not find that miRNAs were downregulated during M2 polarization. Then, we detected the expression of miRNAs that were significantly upregulated during M2 polarization and during M1 polarization. qPCR results showed that the expressions of mmu-miR-5620-5p, mmu-miR-199b-5p, mmu-miR-362-5p and mmu-miR-148a-3p were upregulated during M1 polarization, which was consistent with M2 polarization (**Figure 1L**). All differentially expressed miRNAs were list in Supplementary material S3 and the differentially expressed miRNA were divided into four cluster according to the similar expression pattern (**Figure S2**). The target genes of differentially expressed miRNA analyzed by bioinformatics were listed in Supplementary material S4.PCA showed that the expression of miRNA could distinguish M0, M1 and M2 macrophages (**Figure 1M**). MiR-novel-3-mature mimics and miR-27b-5p mimics were transfected in RAW264.7 cells for overexpressed miR-novel-3-mature and miR-27b-5p in RAW264.7 cells (**Figures 1N, O**). At the same time, RAW264.7 cells were induced into M1 polarization, we found that overexpression of miR-novel-3-nature and miR-27b-5p could promote the mRNA expression of TNF-α which was marker gene of M1 macrophages (**Figure 1P**).

### Changes in piRNA Expression During Macrophage Polarization

**Figure 2:**
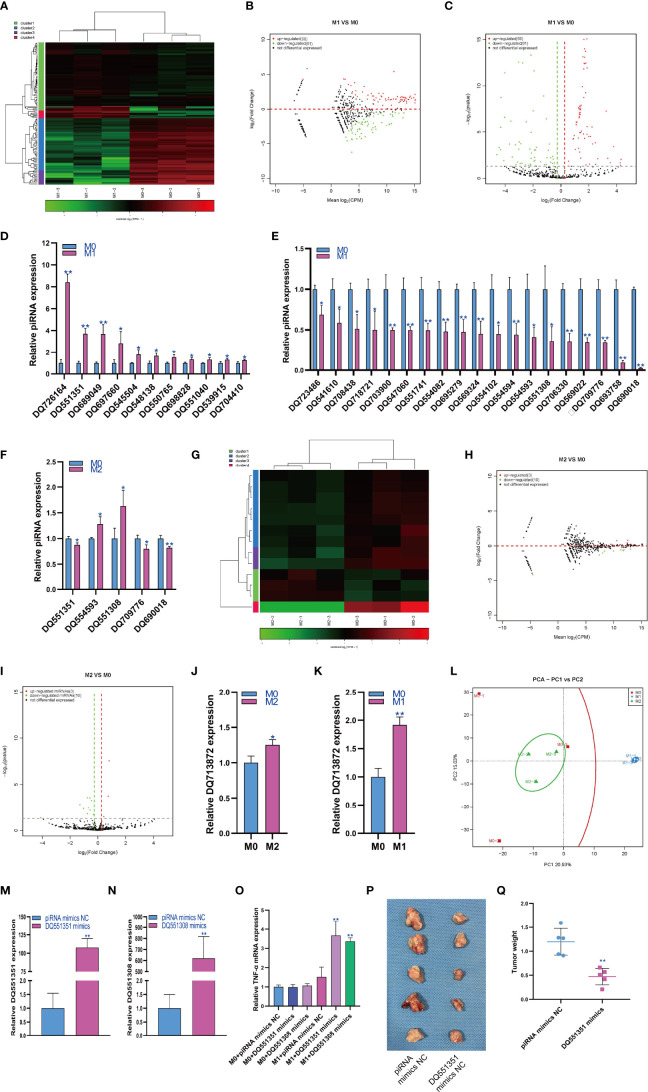
Changes in piRNA expression during macrophage polarization. Hotmap **(A)**, MA **(B)** and volcano plot **(C)** show the small RNA sequencing results of the expression of piRNA in M0 and M1 macrophages. **(D)** The expression of piRNA upregulated in M1 macrophages as detected by qPCR. **(E)** The expression of piRNA was downregulated in M1 macrophages as detected by qPCR. **(F)** The expression of piRNA in M2 macrophages as detected by qPCR. Hotmap **(G)**, MA **(H)** and volcano plot **(I)** show the small RNA sequencing results of the expression of miRNA in M0 and M2 macrophages. **(J)** The expression of piRNA in M2 macrophages as detected by qPCR. **(K)** The expression of piRNA in M1 macrophages as detected by qPCR. **(L)** PCA of piRNA expression in M0, M1 and M2 macrophages. * indicates p < 0.05 compared to M0, and ** indicates p < 0.01 compared to M0. **(M)** The expression of piRNA DQ551351 in RAW264.7 cells transfected with piRNA mimics NC or piRNA DQ551351 mimics were detected by qPCR. **(N)** The expression of piRNA DQ551308 in RAW264.7 cells transfected with piRNA mimics NC or piRNA DQ551308 mimics were detected by qPCR. **(O)** The expression of TNF-α in RAW264.7 cells were detected by qPCR, * indicates p < 0.05 and ** indicates p < 0.01 compared to M1+ miRNA mimics NC group. **(P)** Photographs showed the size of the tumor in mouse injected with RAW264.7 cells with piRNA mimics NC or piRNA DQ551308 mimics. **(Q) **Tumor weight in mouse injected with RAW264.7 cells with piRNA mimics NC or piRNA DQ551308 mimics, ** indicates p < 0.01 compared to piRNA mimics NC group.

The regulatory role of piRNAs in the occurrence and development of diseases has attracted increasing attention. Therefore, we analyzed the expression changes of piRNA during macrophage polarization. The results showed that 69 piRNAs were upregulated and 61 piRNAs were downregulated during M1 macrophage polarization (**Figures 2A–C**). qPCR analysis showed that 11 piRNAs were upregulated and 19 piRNAs were downregulated during M1 polarization (**Figures 2D, E**). Then, we detected the expression of piRNAs with significant differentiation during M1 polarization and during M2 polarization. qPCR results showed that the expression of DQ709776 and DQ690018 was downregulated during M2 polarization, which was consistent with that during M1 polarization. However, the expression of DQ551351 was downregulated and the expressions of DQ554593 and DQ551308 were upregulated during M2 polarization, which was opposite to that during M1 polarization (**Figure 2F**). The results showed that 3 piRNAs were upregulated and 10 piRNAs were downregulated during macrophage M2 polarization (**Figures 2G–I**). We only found that the expression of DQ7138872 was upregulated during M2 polarization using qPCR analysis (**Figure 2J**). On the other hand, we found that DQ7138872 was also upregulated during M1 polarization (**Figure 2K**). The PCA showed that the expression of piRNA could distinguish M0 macrophages from M1 macrophages and M1 macrophages from M2 macrophages but could not distinguish M2 and M0 macrophages (**Figure 2L**). PiRNA DQ551351 mimics and DQ551308 mimics were transfected in RAW264.7 cells for overexpressed DQ551351 and DQ551308 in RAW264.7 cells (**Figures 2M, N**). At the same time, RAW264.7 cells were induced into M1 polarization, we found that overexpression of DQ551351 and DQ551308 could promote the mRNA expression of TNF-α which was marker gene of M1 macrophages (**Figure 2O**). As M1-polarized macrophages showed antitumor activity, we detected the effect of piRNA DQ551351 on the antitumor activity of M1 macrophages, and the result showed that piRNA DQ551351overexpression promoted the antitumor activity of M1 macrophages (**Figures 2P, Q**).

### Changes in snoRNA Expression During Macrophage Polarization

**Figure 3:**
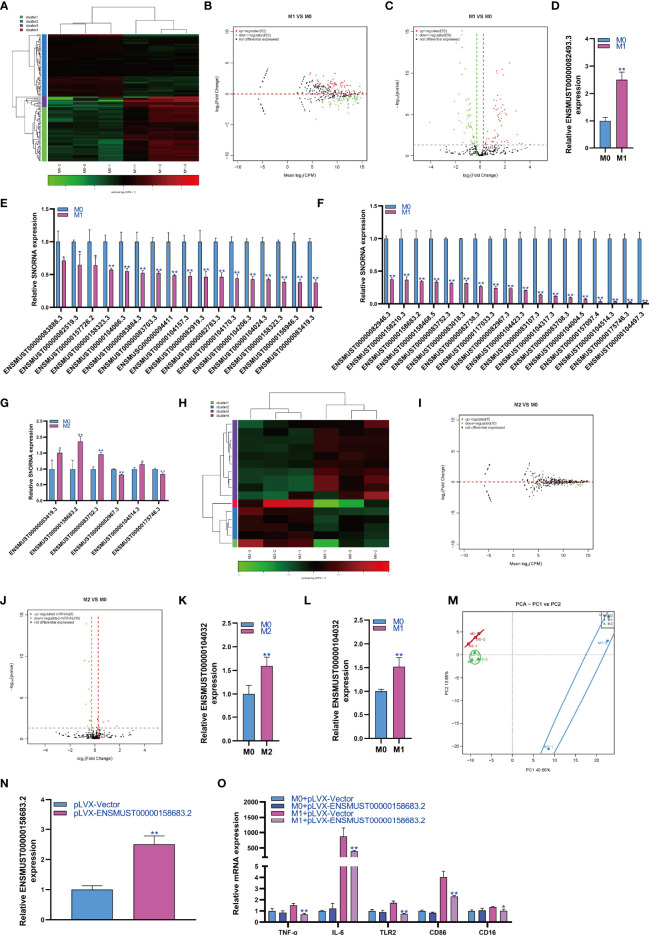
Changes in snoRNA expression during macrophage polarization. Hotmap **(A)**, MA **(B)** and volcano plot **(C)** show the small RNA sequencing results of the expression of snoRNA in M0 and M1 macrophages. **(D)** The expression of snoRNA upregulated in M1 macrophages as detected by qPCR. **(E, F)** The expression of snoRNA was downregulated in M1 macrophages as detected by qPCR. **(G)** The expression of snoRNA in M2 macrophages as detected by qPCR. Hotmap **(H)**, MA **(I)** and volcano plot **(J)** show the small RNA sequencing results of the expression of snoRNA in M0 and M2 macrophages. **(K)** The expression of snoRNA in M2 macrophages as detected by qPCR. **(L)** The expression of piRNA in M1 macrophages as detected by qPCR. **(M)** PCA of snoRNA expression in M0, M1 and M2 macrophages. * indicates p < 0.05 compared to M0, and ** indicates p < 0.01 compared to M0. **(N)** The expression of snoRNA ENSMUST00000158683.2 in RAW264.7 cells transfected with pLVX-Vector or pLVX-ENSMUST00000158683.2 were detected by qPCR. **(O)** The expression of TNF-α, IL-6, TLR2, CD86 and CD16 in RAW264.7 cells were detected by qPCR, * indicates p < 0.05 and ** indicates p < 0.01 compared to M1+ pLVX-Vector group.

SnoRNA is a class of highly conserved molecules among species. Therefore, we analyzed the expression changes of snoRNA during macrophage polarization. The results showed that 62 snoRNAs were upregulated and 59 snoRNAs were downregulated during M1 macrophage polarization (**Figures 3A–C**). qPCR analysis showed that snoRNA ENSMUST00000082493.3 was upregulated during M1 polarization (**Figure 3D**). On the other hand, qPCR analysis showed that 35 snoRNAs were downregulated during M1 polarization (**Figures 3E, F**). Then, we detected the expression of snoRNAs with significant differentiation during M1 polarization and during M2 polarization. qPCR results showed that the expressions of ENSMUST00000083419.3, ENSMUST00000158683.2, ENSMUST00000083752.3 and ENSMUST00000104514.3 were upregulated during M2 polarization, which was contrary to that during M1 polarization. However, the expression of ENSMUST00000082967.3 and ENSMUST00000175746.3 was downregulated during M2 polarization, which was consistent with that during M1 polarization (**Figure 3G**). The results showed that 6 snoRNAs were upregulated and 10 snoRNAs were downregulated during M2 macrophage polarization (**Figures 3H–J**). qPCR analysis showed that only ENSMUST00000104032 was upregulated during M2 polarization (**Figure 3K**). However, we did not find snoRNA downregulation during M2 polarization using qPCR. qPCR results showed that the expression of ENSMUST00000104032 was also upregulated during M1 polarization, which was consistent with that during M2 polarization (**Figure 3L**). PCA showed that the expression of snoRNA could distinguish M0, M1 and M2 macrophages (**Figure 3M**). In order to study the effect of snoRNA on M1 polarization of RAW264.7 cells, we overexpressed snoRNA ENSMUST00000158683.2 in RAW264.7 cells by lentivirus and the qPCR result showed that the expression of snoRNA ENSMUST00000158683.2 in pLVX-ENSMUST00000158683.2 group were higher than pLVX-Vector group (**Figure 3N**). At the same time, RAW264.7 cells were induced into M1 polarization, we found that overexpression of ENSMUST00000158683.2 could inhibit the mRNA expression of TNF-α, IL-6, TLR2, CD86 and CD16 which was marker gene of M1 macrophages (**Figure 3O**).

### Changes in snRNA Expression During Macrophage Polarization

**Figure 4:**
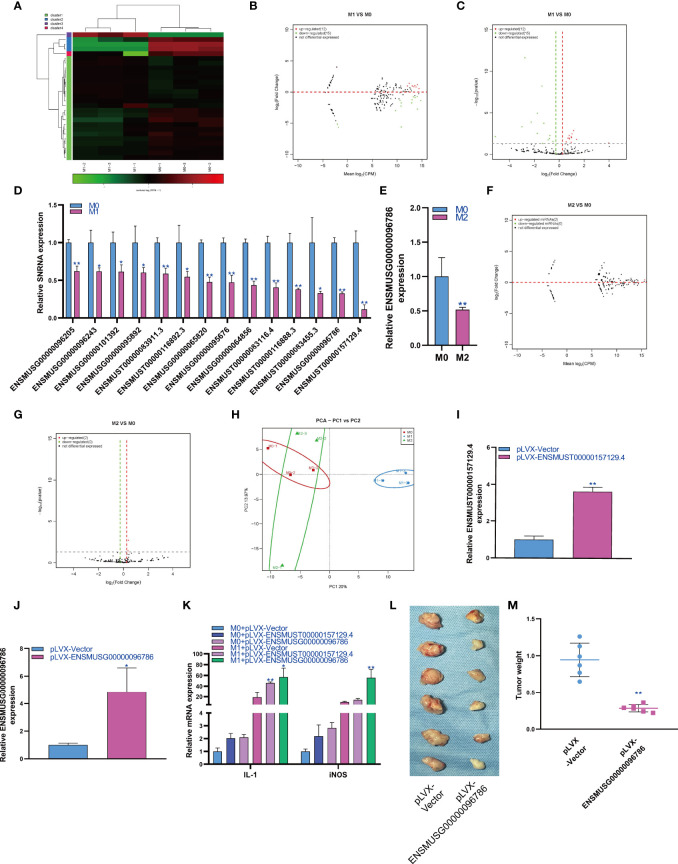
Changes in snRNA expression during macrophage polarization. Hotmap **(A)**, MA **(B)** and volcano plot **(C)** show the small RNA sequencing results of the expression of snRNA in M0 and M1 macrophages. **(D)** The expression of snRNA in M1 macrophages as detected by qPCR. **(E)** The expression of snRNA in M2 macrophages as detected by qPCR. MA **(F)** and volcano **(G)** show the small RNA sequencing results of the expression of snRNA in M0 and M2 macrophages. **(H)** PCA of snRNA expression in M0, M1 and M2 macrophages. * indicates p < 0.05 compared to M0, and ** indicates p < 0.01 compared to M0. **(I)** The expression of snRNA ENSMUST00000157129.4 in RAW264.7 cells transfected with pLVX-Vector or pLVX- ENSMUST00000157129.4 were detected by qPCR. **(J)** The expression of snRNA ENSMUSG00000096786 in RAW264.7 cells transfected with pLVX-Vector or pLVX- ENSMUSG00000096786 were detected by qPCR. **(K)** The expression of IL-1 and iNOS in RAW264.7 cells were detected by qPCR, * indicates p < 0.05 and ** indicates p < 0.01 compared to M1+ pLVX-Vector group. **(L)** Photographs showed the size of the tumor in mouse injected with RAW264.7 cells with pLVX-Vector or pLVX- ENSMUSG00000096786. **(M)** Tumor weight in mouse injected with RAW264.7 cells with pLVX-Vector or pLVX- ENSMUSG00000096786, ** indicates p < 0.01 compared to pLVX-Vector group.

SnRNA can effectively regulate the physiological function of cells by regulating the alternative splicing of mRNA. Therefore, we analyzed the expression changes of snRNA during macrophage polarization. The results showed that 12 snRNAs were upregulated and 15 snRNAs were downregulated during M1 macrophage polarization (**Figures 4A–C**). qPCR analysis showed that 14 snRNAs were downregulated during M1 polarization (**Figure 4D**) and that snRNAs were not upregulated during M1 polarization. Then, we detected the expression of snRNAs that were downregulated during M1 polarization and during M2 polarization. qPCR results showed that the expression of ENSMUST00000104032 was downregulated during M2 polarization, which was consistent with M1 polarization (**Figure 4E**). The results showed that 2 snRNAs were upregulated during macrophage M2 polarization (**Figures 4F, G**). qPCR analysis did not find the differential expression of snRNA during M2 polarization. The PCA showed that the expression of snRNA could distinguish M0 macrophages from M1 macrophages and M1 macrophages from M2 macrophages but could not distinguish between M2 and M0 macrophages (**Figure 4H**). In order to study the effect of snRNA on M1 polarization of RAW264.7 cells, we overexpressed snRNA ENSMUST00000157129.4 and ENSMUSG00000096786 in RAW264.7 cells by lentivirus and the qPCR result showed that the expression of snRNA ENSMUST00000157129.4 and ENSMUSG00000096786 in pLVX-ENSMUST00000157129.4 and pLVX-ENSMUSG00000096786 group were higher than pLVX-Vector group (**Figures 4I, J**). At the same time, RAW264.7 cells were induced into M1 polarization, we found that overexpression of ENSMUST00000157129.4 and ENSMUSG00000096786 could promote the mRNA expression of IL-1 and iNOS which was marker gene of M1 macrophages (**Figure 4K**). We detected the effect of ENSMUSG00000096786 on the antitumor activity of M1 macrophages, and the result showed that ENSMUSG00000096786 overexpression promoted the antitumor activity of M1 macrophages (**Figures 4L, M**).

### Changes in Repeat RNA Expression During Macrophage Polarization

**Figure 5:**
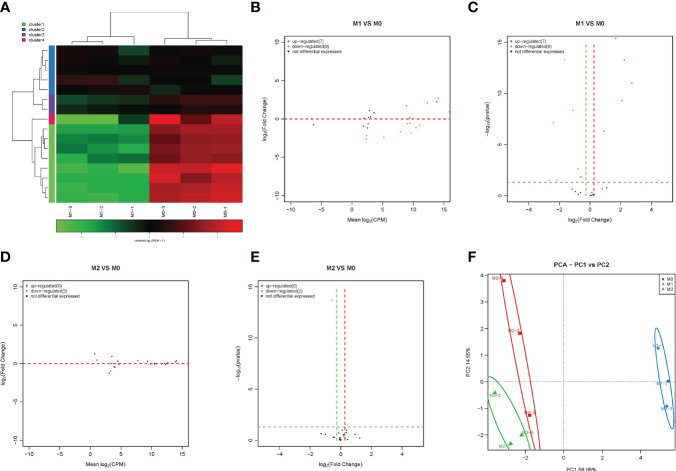
Changes in repeat RNA expression during macrophage polarization. Hotmap **(A)**, MA **(B)** and volcano plot **(C)** show the small RNA sequencing results for the expression of repeat RNA in M0 and M1 macrophages. MA **(D)** and volcano **(E)** show the small RNA sequencing results for the expression of repeat RNA in M0 and M2 macrophages. **(F)** PCA of repeat RNA expression in the M0, M1 and M2 macrophages.

Repeat RNA is a kind of very special RNA molecule with repetitive sequences. Therefore, we analyzed the expression changes of repeat RNA during macrophage polarization. The results showed that 7 repeat RNAs were upregulated and 9 repeat RNAs were downregulated during M1 macrophage polarization (**Figures 5A** **–C**). On the other hand, the results showed that 2 repeat RNAs were downregulated during macrophage M2 polarization (**Figures 5D, E**). PCA showed that the expression of repeat RNA could distinguish M0, M1 and M2 macrophages (**Figure 5F**).

## Discussion

As an important part of the innate immune system, macrophages have the functions of phagocytosis and killing pathogenic microorganisms and immune information transmission. They play an important role in inflammatory defense and tissue development and in maintaining the dynamic balance of the body. Resting macrophages can undergo morphological, functional and phenotypic differentiation under the action of different microenvironment signals *in vivo* and *in vitro*, that is, macrophage polarization (36). According to the difference in their immunological function, polarized macrophages can be divided into M1 macrophages with the classical activation pathway and into M2 macrophages with the alternative activation pathway (37). Macrophage polarization is controlled by many molecules, such as signaling pathways, transcription factors, epigenetic mechanisms and posttranscriptional regulatory factors, which play a corresponding role in the process of changes in the body’s microenvironment and affect disease progression and outcome (38). This functional transformation is closely related to the selective gene expression regulation of signaling pathways.

The small noncoding RNAome has received increasing attention in the regulation of cell function. However, the changes in the small noncoding RNAome under normal physiological and pathological conditions need to be further discussed. Panagiotis and his colleagues reported expression changes in the small noncoding RNAome in equine and human chondrocytes during aging (39). Michelle reported the expression changes of the small noncoding RNAome in the plasma of patients with rheumatoid arthritis and found that the contents of miRNA and TDRS in the plasma of RA patients increased significantly, while the contents of yDRS decreased significantly (40). Their research mainly focused on the expression changes of miRNAs, snoRNAs, snRNAs and tRNAs. In our study, we mainly focused on the expression changes of miRNAs, piRNAs, snoRNAs, snRNAs and repeat RNAs during macrophage polarization.

MiRNAs have been considered important regulators of macrophage function, and part of their role is achieved by regulating the polarization of macrophages. Cho et al. found that the production of miRNAs in macrophages is necessary for the anti-inflammatory polarization of macrophages by using macrophage-specific Disher gene knockout (41). Banerjee et al. found that the expression of miR-125a-5p in M2 macrophages was higher than that in M1 macrophages and confirmed that miR-125a-5p could reduce the expression of the M1 phenotype induced by LPS and promote the expression of M2 induced by IL-4 by targeting KLF13 to regulate the phagocytosis and bactericidal activity of macrophages (42). Li et al. found that exosomes derived from bone marrow mesenchymal stem cells containing miR-124-3p can mediate the expression of Ern1 in macrophages and induce M2 phenotypic polarization of macrophages (43). In addition, a large number of studies have confirmed that many kinds of miRNAs can affect the polarization process of macrophages to M1 or M2. MiR-27a, miR-27b, miR-130a, miR-130b, miR-155, miR-21 and miR-26a could promote M1 polarization and inhibit M2 polarization to promote inflammation (44–47). MiR-9, miR-146a, miR-146b, miR-124, Let-7c and miR-210 could inhibit M1 polarization and promote M2 polarization to inhibit inflammation (38, 48–50). In our study, we also found that the expression of miR-155 increased during macrophage polarization to M1. At the same time, we also found that multiple new miRNAs may be involved in regulating macrophage polarization.

PiRNAs are a class of ncRNAs first found and identified in Drosophila testes (51). Its structure, biosynthetic process and partial regulation have gradually become clear in recent years. At the same time, it has been proven to play an important role in some pathophysiological processes, especially in tumorigenesis. Yin et al. reported that the expression of piR-823, piR-18849 and piR-19521 increased in the serum and tissues of patients with colorectal cancer, suggesting that they may be related to the pathogenesis of this kind of cancer and can be used as diagnostic markers of colorectal cancer (52, 53). Stohr et al. found that the expression of PIWIL1, PIWIL2 and PIWIL4 decreased in patients with renal cell carcinoma and considered that they could be used as prognostic biomarkers of this kind of cancer (54). Han et al. confirmed that piRNA-30473 can trigger the downstream signal cascade by regulating m6A RNA methylation, resulting in the occurrence and adverse prognosis of diffuse large B-cell lymphoma (55). We found that the expression of piRNA changed significantly during macrophage polarization. However, no study has reported the regulatory effect of piRNAs on macrophage polarization, so we will further study the regulatory effect of piRNAs on macrophage polarization.

SnoRNA is a kind of noncoding RNA mainly derived from introns, with a length of approximately 50-250 nucleotides (56). SnoRNA plays an important role in cell development, homeostasis and many diseases. Ellen et al. demonstrated for the first time that snoRNA is involved in the biology of articular chondrocytes. Under different arthritis conditions, the expression level of U3 snoRNA in articular chondrocytes is reduced, significantly reducing the protein translation ability of the cell (57). Mandy et al. found abnormal expression of the snoRNA genome in aging cartilage and diseased tendons, in which the expression of SNORD26 increased in osteoarthritis, while SNORD44 and SNORD78 decreased with age (58). At present, the regulatory effect of snoRNA on macrophage polarization has not been reported. In this study, we also reported the expression changes of snoRNA during macrophage polarization for the first time. Our results show that the expression of snoRNA is significantly different in the process of macrophage polarization, which may be involved in the regulation of macrophage polarization.

Small nuclear RNAs are a class of noncoding RNAs with a length of 100-215 nucleotides that widely exist in the nucleus of eukaryotes and mainly include the U1, U2, U3, U4, U5, U6 and U11 genes (29). Increasing evidence has shown that snRNA is related to the regulation of gene expression in different types of tumors. Wang et al. found that U11 may be involved in the regulation of gene expression in bladder cancer cells, which can alter gene expression by affecting the PI3K-Akt signaling pathway and may be a potential biomarker for the clinical diagnosis and treatment of bladder cancer (28). In other systems, Cheng et al. found that abnormalities in U11 snRNA in neuronal cells significantly interfere with RNA splicing, thus damaging nerve cell function and affecting hippocampal synaptic plasticity and spatial learning and memory ability (59). This study also reported the expression changes of snRNA during macrophage polarization for the first time. Moreover, we found that the expression of multiple snRNAs decreased during macrophage polarization to M1 but did not find that the expression of snRNAs increased during macrophage polarization to M1 and M2.

Sequencing results showed that repeat RNA was differentially expressed during M1 and M2 macrophage polarization. However, due to the rare research on repeat RNA and its short length, it has been difficult to verify the sequencing results by conventional real-time PCR. More studies are needed to explore the role of repeat RNA in chondrocyte aging. This study also reported the expression changes of repeat RNA during macrophage polarization for the first time, and the function of repeat RNA needs further study.

## Conclusion

Our results show that the expression of miRNA, piRNA, snoRNA, snRNA and repeat RNA changes during the polarization of macrophages to M1 or M2. At the same time, there are no reports on the role of piRNA, snoRNA and snRNA in the process of macrophage polarization, but our results preliminarily show that the expression of these sncRNAs is significantly upregulated or downregulated during macrophage polarization, suggesting that they may be regulated and changed in this process, which may play an important role in the process of macrophage polarization. Overexpression of miRNA miR-novel-3-nature and miR-27b-5p and piRNA DQ551351 and DQ551308 could promote the mRNA expression of TNF-α which was marker gene of M1 macrophages. Overexpression of snoRNA ENSMUST00000158683.2 could inhibit the mRNA expression of TNF-α. Overexpression of snRNA ENSMUST00000157129.4 and ENSMUSG00000096786 could promote the mRNA expression of IL-1 and iNOS which was marker gene of M1 macrophages. piRNA DQ551351 and snRNA ENSMUSG00000096786 overexpression promoted the antitumor activity of M1 macrophages. In view of these significant differences in sncRNA, further research is still needed in the future to explore its action targets and mechanisms to better analyze the process of macrophage polarization and deepen the understanding of various pathophysiological changes.

## Data Availability Statement

The datasets presented in this study can be found in online repositories. The names of the repository/repositories and accession number(s) can be found below: GEO - GSE198744.

## Ethics Statement

The animal study was reviewed and approved by Xinhua Hospital Affiliated to Shanghai Jiao Tong University School of Medicine.

## Author Contributions

Conceptualization: XiaZ and CW; methodology: DM, JY, and XZ; software: YW, JP, and CW; data curation: LD and CW; writing, review, and editing: CW. All authors contributed to the article and approved the submitted version.

## Funding

This work was supported by the National Natural Science Foundation of China (81802191), Shanghai Sailing Program (21YF1443300) and Interdisciplinary Medicine and Engineering Foundation of Shanghai Jiao Tong University (YG2019QNB30). Shanghai Sailing Program (19YF1431200).

## Conflict of Interest

The authors declare that the research was conducted in the absence of any commercial or financial relationships that could be construed as a potential conflict of interest.

## Publisher’s Note

All claims expressed in this article are solely those of the authors and do not necessarily represent those of their affiliated organizations, or those of the publisher, the editors and the reviewers. Any product that may be evaluated in this article, or claim that may be made by its manufacturer, is not guaranteed or endorsed by the publisher.

## Supplementary Material

Supplementary Figure 1 Macrophage polarization. **(A)** The iNOS mRNA expression during M1 polarization. **(B)** The CD206 mRNA expression during M2 polarization. ** indicates p < 0.01 compared to M0 group.

Supplementary Figure 2 The cluster of different expressed miRNA during macrophage polarization. The different expressed miRNA were divided into four cluster according to the similar expression pattern among M1 **(A)** and M2 **(B)** macrophage polarization.
