# Supplementary figures and images for: Changes in the Small Noncoding RNAome During M1 and M2 Macrophage Polarization
Source: Front Immunol. 2022 May 10;13:799733. doi: 10.3389/fimmu.2022.799733 (PMC9127141; doi:10.3389/fimmu.2022.799733)

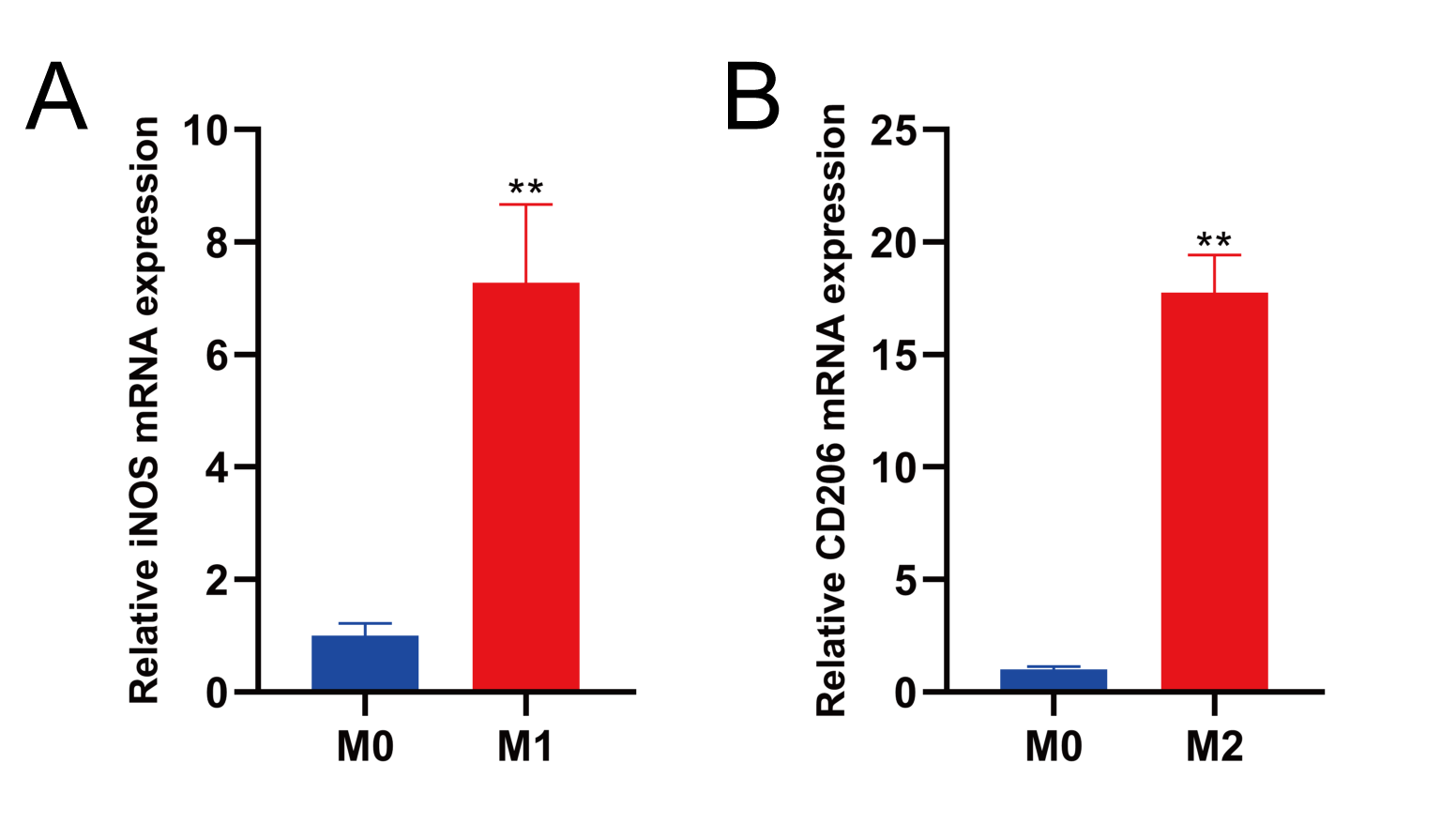

Supplement: Supplementary Figure 1 — Macrophage polarization. (A) The iNOS mRNA expression during M1 polarization. (B) The CD206 mRNA expression during M2 polarization. ** indicates p < 0.01 compared to M0 group. [file Image_1.tif]

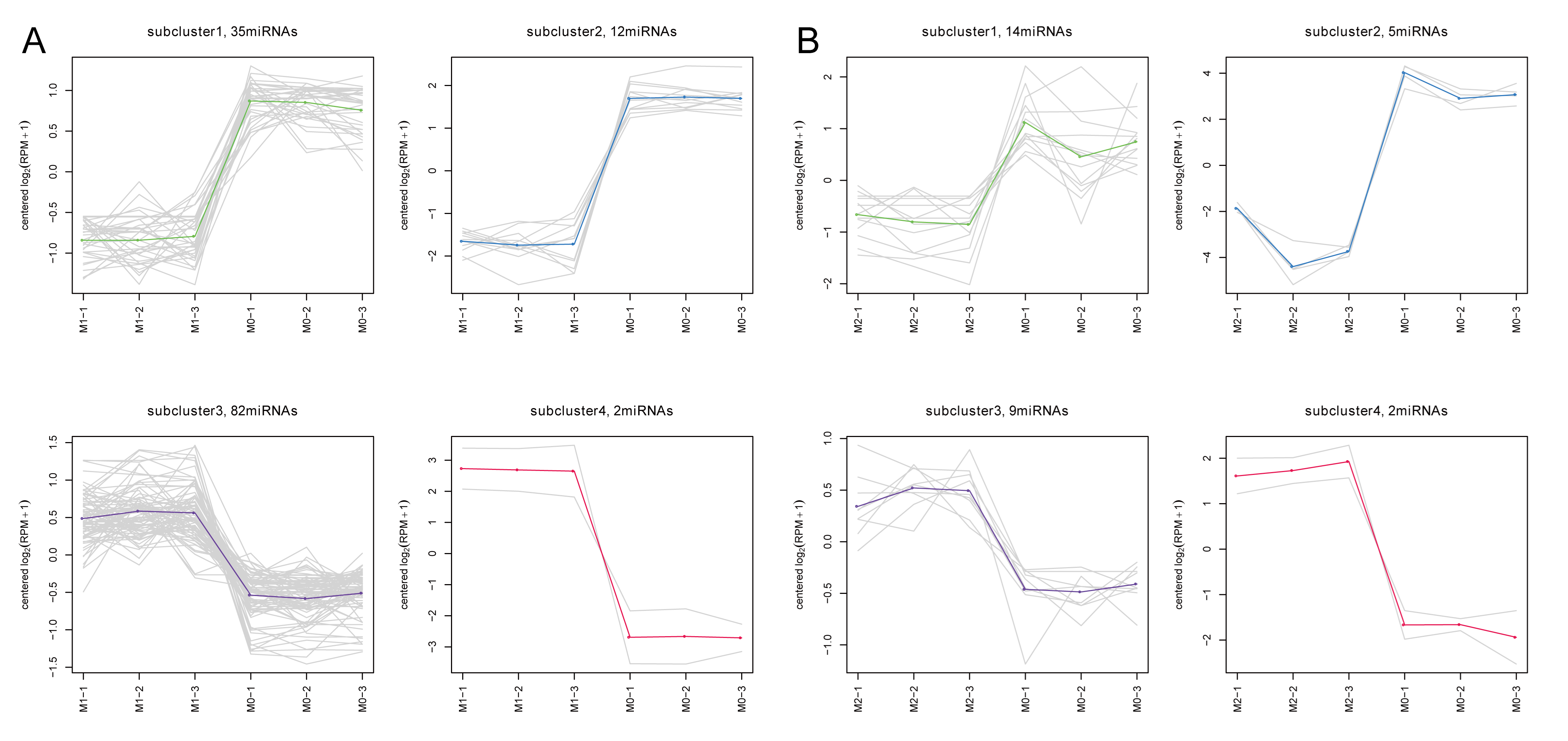

Supplement: Supplementary Figure 2 — The cluster of different expressed miRNA during macrophage polarization. The different expressed miRNA were divided into four cluster according to the similar expression pattern among M1 (A) and M2 (B) macrophage polarization. [file Image_2.tif]
